# The importance of study duration and spatial scale in pathogen detection—evidence from a tick-infested island

**DOI:** 10.1038/s41426-018-0188-9

**Published:** 2018-11-28

**Authors:** Jani Jukka Sormunen, Tero Klemola, Jari Hänninen, Satu Mäkelä, Ilppo Vuorinen, Ritva Penttinen, Ilari Eerikki Sääksjärvi, Eero Juhani Vesterinen

**Affiliations:** 1 Department of Biology, University of Turku, FI-20014 Turku, Finland; 2 Biodiversity Unit, University of Turku, FI-20014 Turku, Finland; 3 Deparment of Agricultural Sciences, University of Helsinki, FI-00014 Helsinki, Finland

## Abstract

Ticks (Acari: Ixodoidea) are among the most common vectors of zoonotic pathogens worldwide. While research on tick-borne pathogens is abundant, few studies have thoroughly investigated small-scale spatial differences in their occurrence. Here, we used long-term cloth-dragging data of *Ixodes ricinus* and its associated, known and putative pathogens (*Borrelia burgdorferi* s.l., *Borrelia miyamotoi*, *Anaplasma phagocytophilum*, *Rickettsia* spp., *Candidatus* Neoehrlichia mikurensis, *Bartonella* spp., *Babesia* spp., and tick-borne encephalitis virus, TBEV) from a small, well-studied island in southwestern Finland to analyze potential temporal and spatial differences in pathogen prevalence and diversity between and within different biotopes. We found robust evidence indicating significant dissimilarities in *B*. *burgdorferi* s.l., *A*. *phagocytophilum*, *Rickettsia*, and *Ca*. N. mikurensis prevalence, even between proximal study areas on the island. Moreover, during the 6 years of the ongoing study, we witnessed the possible emergence of TBEV and *Ca*. N. mikurensis on the island. Finally, the stable occurrence of a protozoan pathogen that has not been previously reported in Finland, *Babesia venatorum*, was observed on the island. Our study underlines the importance of detailed, long-term tick surveys for public health. We propose that by more precisely identifying different environmental factors associated with the emergence and upkeep of enzootic pathogen populations through rigorous longitudinal surveys, we may be able to create more accurate models for both current and future pathogen distributions.

## Introduction

Ticks (Acari: Ixodoidea) are among the most important transmitters of zoonotic pathogens of medical interest^1–3^. In northern Europe, the geographical distribution of *Ixodes ricinus* (the sheep tick), the major European tick-borne pathogen (TBP) vector, has shifted northwards in recent decades^4,5^. Furthermore, tick abundance seems to be rising in established tick areas^4,6^. These changes have mostly been attributed to various effects of climate change^4^. In addition to increasing tick distribution and abundance, changes in climatic conditions can facilitate the spread and amplification of TBPs^7^.

In southern Finland, *Ixodes ricinus* ticks serve as the primary vectors for tick-borne encephalitis virus (TBEV) and *Borrelia burgdorferi* sensu lato, the bacterial group responsible for Lyme borreliosis (LB). In addition to these two pathogens of considerable medical interest, European *I. ricinus* have also been found to carry several other bacterial and protozoan pathogens, whose importance as zoonotic agents has not yet been fully established: *Rickettsia* spp. (causing spotted fever), *Borrelia miyamotoi* (relapsing fever), *Anaplasma phagocytophilum* (human granulocytic anaplasmosis), *Babesia* spp. (babesiosis), *Candidatus* Neoehrlichia mikurensis (neoehrlichiosis), and *Bartonella* spp. (cat scratch fever, bartonellosis)^8–12^. In Finland, *B. burgdorferi* s.l. and TBEV have been reported from ticks in several studies over the past few decades^5,13–15^. In contrast, *Rickettsia helvetica*, *R. monacensis*, and *A. phagocytophilum* have only recently been reported^6,16^. While the medical impact of these recently detected pathogens is expected to be low compared to LB and TBE, knowledge of their occurrence may nevertheless prove important, as co-infections with different pathogens can cause unpredictable, more severe diseases^17,18^.

Recently published tick-related studies from the Nordic countries have mostly focused on assessing changes in the distribution of *I. ricinus* and *I. persulcatus* and mapping TBP occurrence^4,5,19–21^. Consequently, whereas longitudinal studies of tick populations and the occurrence of associated pathogens at specific locations have been published elsewhere in Europe (e.g.,^22–24^), such data from Fennoscandia are rare. Localities found to harbor tick populations and TBPs should be monitored for longer periods of time to determine whether the detected pathogens are indeed established; whether they form stable, increasing, or decreasing populations; and to detect the possible emergence of novel pathogens. Indeed, a longitudinal survey conducted in a French suburban forest revealed dramatic fluctuations in the annual prevalence of some TBPs, despite little measured variation in abiotic conditions (data from a weather station)^25^. These results suggest that localized, limited-scale changes in, for example, host animal communities (as suggested by the authors in^25^) may affect tick and pathogen occurrence, even in relatively stable climate conditions.

As no vaccines or other preventive cures yet exist against tick-borne diseases apart from TBEV, human protection must rely mostly on preventive measures: increasing awareness and helping citizens avoid risk areas. Avoiding risk areas requires knowledge of such localities, which is mostly gathered by sampling ticks via cloth dragging and/or, to an increasing degree, predictive modeling with Geographical Information Systems (GIS)^26–28^. However, ticks are not equally distributed in every biotope; rather, they tend to aggregate to certain biotopes^29–31^. As research projects seldom have unlimited time and resources, sampling may be limited to such primary tick biotopes. Consequently, if the environmental conditions most beneficial to each TBP differ from those of the tick hosts (or other pathogens), the results of such spot-checks can convey an incomplete view of the occurrence and environmental preferences of TBPs. This is particularly problematic when such data are used as a basis for projection in distribution modeling^28^. Consequently, longitudinal surveys regarding the prevalence of a wide range of TBPs in different biotopes and/or areas of close proximity are needed to assess the environmental preferences of TBPs and the degree to which their occurrence in tick populations fluctuates across space and time.

In the current study, we examined longitudinal patterns in tick-borne pathogen diversity and prevalence by screening *I. ricinus* collected over 6 years from a small island (surface area 1.6 km^2^; coordinates 60°14’4”N, 21°57’7”E) for eight different pathogens or pathogen groups. Furthermore, we assessed whether differences in the prevalence of the most common pathogens could be detected among nymph communities in different biotopes and fixed study transects on the island. We aimed to increase awareness on the importance of longitudinal studies of tick populations regarding the occurrence and emergence of pathogens, as well as the small geographical scale at which pathogen prevalence may vary.

## Results

In total, 182 adult *I. ricinus*, 2370 nymphs, and 4518 larvae collected in Seili from 2012–2017 were screened for the listed pathogens. Analyzed samples consisted of 182 individual adults, 1950 individual nymphs, 123 nymph pools (420 nymphs, 2–14 per pool; DNA/RNA was extracted from nymph pools in 2012 but from individual nymphs in 2013–2017; pooled samples were not used in prevalence calculations), and 313 larvae pools (4518 larvae; 1-111 per pool). Apart from *Bartonella*, all screened pathogens were detected during the study period (Tables 1, 2).

**Table 1:** Annual prevalence of tick-borne pathogens in nymph and adult *Ixodes ricinus* from Seili Island

	Study year						
	2012	2013	2014	2015	2016	2017	Total^a^
Nymphs analyzed	437	227	400	275	535	495	1932
Adults analyzed	44	19	27	38	35	19	182
Pathogen species/groups:							
*Borrelia burgdorferi* sensu lato							
Nymphs (prevalence)	30 (6.7)^b^	37 (16.3)	76 (19.0)	48 (17.5)	93 (17.4)	119 (24.0)	373 (19.3)
Adults (prevalence)	11 (25.0)	5 (26.3)	9 (33.3)	5 (13.2)	6 (17.1)	10 (52.6)	46 (25.3)
*Borrelia miyamotoi*							
Nymphs	2 (0.5)^b^	3 (1.3)	1 (0.3)	2 (0.7)	3 (0.6)	4 (0.8)	13 (0.7)
Adults	0	0	1 (3.7)	1 (2.6)	0	0	2 (1.1)
*Anaplasma phagocytophilum*							
Nymphs	4 (0.9)^b^	11 (4.9)	6 (1.5)	7 (2.5)	23 (4.3)	18 (3.6)	65 (3.4)
Adults	2 (4.5)	3 (15.8)	3 (11.1)	2 (6.3)	6 (17.1)	2 (10.5)	18 (9.9)
*Rickettsia* spp.							
Nymphs	17 (3.9)^b^	4 (1.8)	9 (2.3)	1 (0.4)	10 (1.9)	12 (2.4)	36 (1.9)
Adults	1 (2.3)	0	0	3 (7.9)	2 (5.7)	0	6 (3.3)
*Babesia* spp.							
Nymphs	1 (0.2)^b^	3 (1.3)	7 (1.8)	2 (0.7)	9 (1.7)	4 (0.8)	25 (1.3)
Adults	0	0	2 (7.4)	0	0	0	2 (1.1)
*Candidatus* Neoehrlichia mikurensis							
Nymphs	0	0	0	5 (1.8)	23 (4.3)	9 (1.8)	37 (1.9)
Adults	0	0	0	0	0	0	0
Tick-borne encephalitis virus							
Nymphs	0	0	0	0	3 (0.6)	4 (0.8)	7 (0.4)
Adults	0	0	0	0	0	0	0
*Bartonella* spp.							
Nymphs	0	0	0	0	0	0	0
Adults	0	0	0	0	0	0	0

^a^2012 nymph samples were excluded from calculations

^b^Most nymph samples in 2012 were pooled; the number in brackets is the minimum infection rate

**Table 2:** Annual minimum infection rates (MIR) for tick-borne pathogens detected in *I. ricinus* larvae from Seili Island

	Study year						
	2012	2013	2014	2015	2016	2017	Total
Larvae analyzed^a^	1281	374	635	342	575	1311	4518
Pathogen species^b^:							
*Rickettsia* spp.							
Positive samples (MIR^c^)	14 (1.1)	2 (0.5)	0 (0)	1 (0.3)	4 (0.7)	16 (1.2)	37 (0.8)
*Babesia* spp.							
Positive samples (MIR^c^)	0 (0)	2 (0.5)	2 (0.3)	1 (0.3)	4 (0.7)	5 (0.4)	14 (0.3)
*Borrelia miyamotoi*							
Positive samples (MIR^c^)	0 (0)	0 (0)	0 (0)	0 (0)	1 (0.2)	2 (0.2)	3 (0.1)

^a^Larvae samples were pools of 1–111 (years 2012–2014) or 1–24 (years 2015–2017) larvae

^b^All larvae samples were negative for *B. burgdorferi* s.l., *Bartonella* spp., *C*. N. mikurensis, *A. phagocytophilum*, and TBEV

^c^Minimum infection rate (estimated one positive individual per positive pool)

*Borrelia burgdorferi* s.l. and *A. phagocytophilum* were the most commonly detected pathogens, with prevalence rates of 19.3% and 3.4% for nymphs and 25.3% and 9.9% for adults, respectively (Table 1). Both pathogens were found from nymphs and adults annually. No positive samples were detected among larvae.

*Rickettsia*, *Babesia*, *Ca*. N. mikurensis, *B. miyamotoi*, and TBEV were detected less frequently, with respective prevalence rates of 1.9, 1.3, 1.9, 0.7, and 0.4% for nymphs and 3.3, 1.1, 0, 1.1, and 0% for adults (Table 1). *Candidatus* N. mikurensis was only detected in 2015–2017 and TBEV in 2016–2017. *Rickettsia, Babesia*, and *B. miyamotoi* were also detected from larvae pools, with minimum infection rates of 0.8%, 0.3%, and 0.1%, respectively (Table 2).

In total, 41 *Rickettsia* and 22 *Babesia* qPCR-positive samples were successfully sequenced. For *Rickettsia*, two species were identified: *R. helvetica* (39/41) and *R. monacensis* (2/41). All *Babesia* samples were identified as *B. venatorum*. Sequenced samples displayed 99–100% identity matches to reference sequences. Representative sequences have been deposited in GenBank (Accession numbers: MH230182, MH230183, and MH256660).

Several individual nymphs and adults were co-infected by two pathogens: 1.9% of nymphs (36 samples) and 3.8% of adults (7 samples), with an overall co-infection rate of 2%. No co-infections by three or more pathogens were detected. The most common co-infections were *B. burgdorferi* s.l. and *Ca*. N. mikurensis for nymphs (0.8% of individual nymph samples) and *B. burgdorferi* s.l. and *A. phagocytophilum* for adults (1.6% of adult samples). Interestingly, a high portion (16/37) of *Ca*. N. mikurensis detections were from samples co-infected with *B. burgdorferi* s.l., which was considerably more often than expected by random co-occurrence (7.1 expected, 16 observed; *χ*^2^ = 13.9, *p* = 0.0002, df = 1). Co-infection types and the associated prevalences are reported in Table 3.

**Table 3:** Co-infections and associated prevalence detected in individual *I. ricinus* nymphs and adults from Seili Island

Year	Life stage^a^	Pathogen pairs						
		Bbsl^b^ & CNe^b^	Bbsl^b^ & Rickettsia	Bbsl^b^ & TBEV^b^	Bbsl^b^ & Babesia	Bbsl^b^ & Anaplasma	Anaplasma & Rickettsia	Babesia & CNe^b^
2012	N	0	1 (0.2)	0	0	0	0	0
	A	0	1 (2.3)	0	0	0	0	0
2013	N	0	1 (0.4)	0	0	1 (0.4)	1 (0.4)	0
	A	0	0	0	0	0	0	0
2014	N	0	1 (0.3)	0	1 (0.3)	0	0	0
	A	0	0	0	2 (7.4)	1 (3.7)	0	0
2015	N	4 (1.5)	1 (0.4)	0	0	0	0	0
	A	0	1 (2.6)	0	0	0	0	0
2016	N	9 (1.7)	1 (0.2)	1 (0.2)	3 (0.6)	0	2 (0.4)	1 (0.2)
	A	0	0	0	0	0	0	0
2017	N	3 (0.6)	0	3 (0.6)	1 (0.2)	1 (0.2)	0	0
	A	0	0	0	0	2 (10.5)	0	0
Total	N	16 (0.8)	5 (0.3)	4 (0.2)	5 (0.3)	2 (0.1)	3 (0.2)	1 (0.1)
	A	0	2 (1.1)	0	2 (1.1)	3 (1.6)	0	0

*N* nymphs, *A*  adults, *Bbsl* *Borrelia burgdorferi* sensu lato, *CNe* *Candidatus* Neoehrlichia mikurensis, *TBEV* tick-borne encephalitis virus

^a^*N*, *A*

^b^Bbsl,CNe, TBEV

**Fig. 1:**
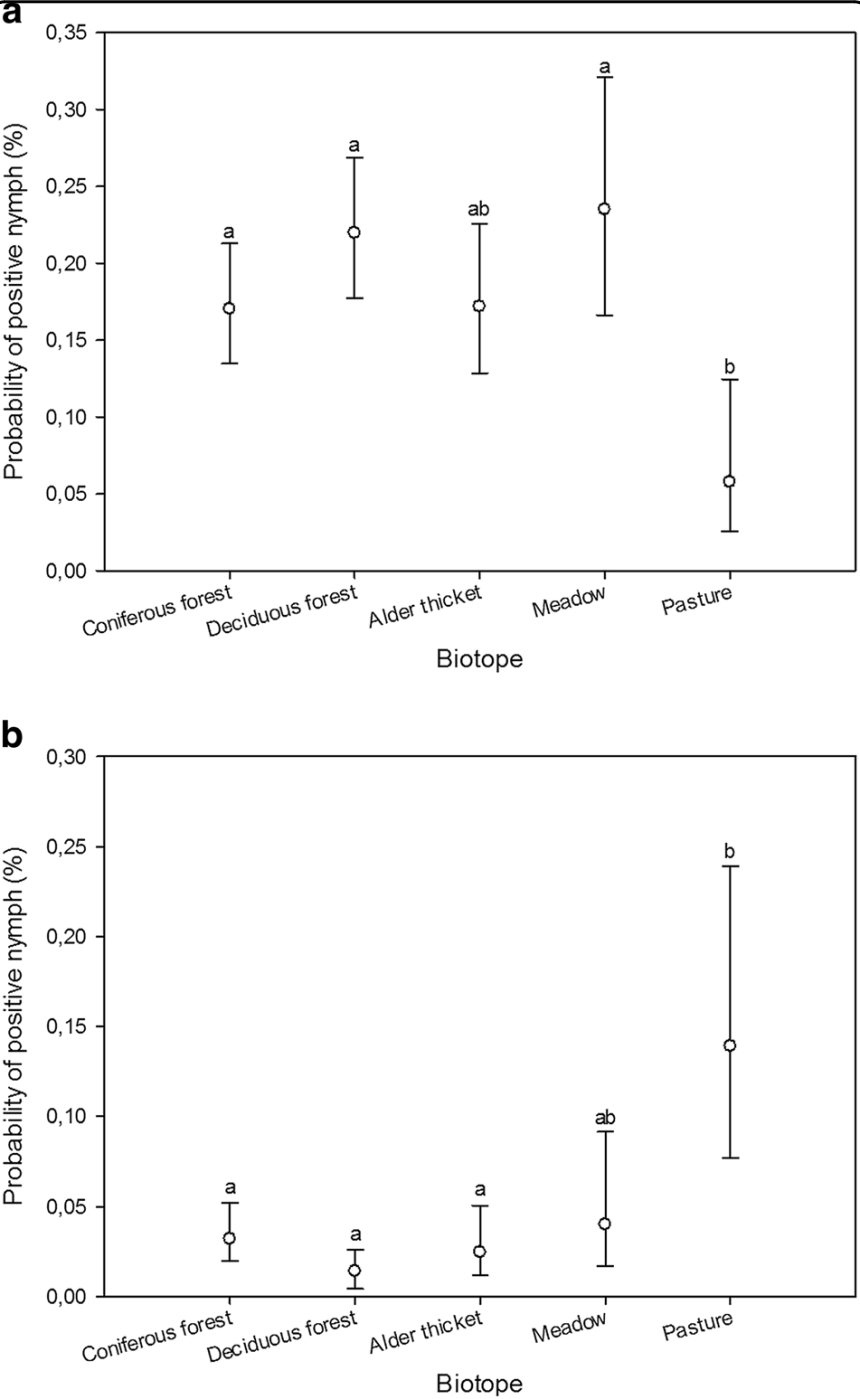
Estimated probabilities (with 95% confidence limits) of nymph samples being positive for *B. burgdorferi* s.l. (**a**) and *A*. *phagocytophilum* (**b**) across biotopes, as predicted by the GLMM. Different biotope classes were assigned matching letters when no statistically significant differences between them could be identified (*p* > 0.05; multiple pairwise comparisons adjusted by Tukey test). Mismatching letters denote statistically significant differences between biotope classes with different letters (*p* < 0.05)

**Fig. 2:**
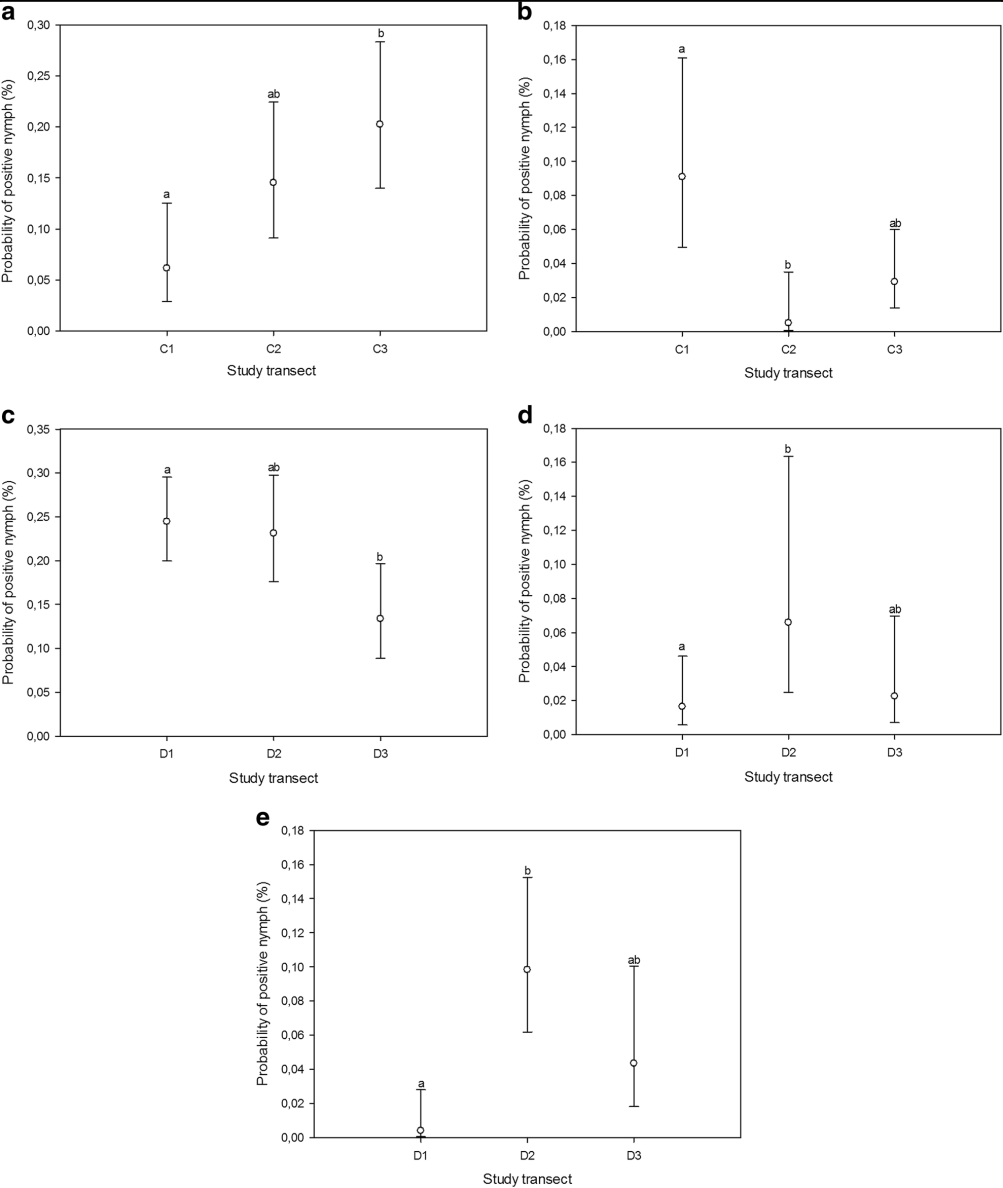
Estimated probabilities (with 95% confidence limits) of nymph samples being positive for *B. burgdorferi* s.l. (**a**, **c**), *A*. *phagocytophilum* (**b**), *Rickettsia* (**d**), and *Ca*. N. mikurensis (**e**) within biotopes (across transects), as predicted by the GLMM. Different transects were assigned matching letters when no statistically significant differences between them could be identified (*p* > 0.05; multiple pairwise comparisons adjusted by Tukey test). Mismatching letters denote statistically significant differences between transects with different letters (*p* < 0.05). C_1-3_, transects in coniferous forest; D_1-3_, transects in deciduous forest

Using nymph samples, differences in prevalence among biotopes were detected for *B. burgdorferi* s.l. (GLMM, *n* = 1932, *F*_4, 1927_ = 4.58, *p* < 0.002) and *A. phagocytophilum* (GLMM, *n* = 1932, *F*_4, 1927_ = 9.44, *p* < 0.0001) (Fig. 1). No definite inter-annual patterns regarding the prevalence of *B. burgdorferi* s.l., *A. phagocytophilum*, or *Rickettsia* could be discerned from BLUP estimates (Technical Appendix: Fig. 1). Intra-biotope analyses revealed differences in *B. burgdorferi* s.l. and *A. phagocytophilum* prevalence among transects in coniferous forest (GLMM, *n* = 548, *F*_2, 545_ = 5.74, *p* < 0.004; GLMM, *n* = 548, *F*_2, 545_ = 5.66, *p* < 0.004) and between *B. burgdorferi* s.l., *Rickettsia*, and *Ca*. N. mikurensis prevalence among transects in deciduous forest (GLMM, *n* = 654, *F*_2, 651_ = 3.9, *p* = 0.02; GLMM, *n* = 654, *F*_2, 651_ = 5.19, *p* < 0.006; GLMM, *n* = 654, *F*_2, 534_ = 5.92, *p* < 0.003) (Fig. 2).

## Discussion

The longitudinal data presented here reveal the persistent occurrence of several tick-borne pathogens in the *I. ricinus* population on Seili Island, with most screened pathogens being detected annually. Along with previously known pathogens^6,13^, the potential emergence of novel microbes was observed in the study area. Furthermore, differences in the occurrence of the most commonly detected pathogens, *B. burgdorferi* s.l. and *A. phagocytophilum*, were found among biotopes and study transects (i.e., specific areas within similar biotopes). These results emphasize the importance of longitudinal studies of tick populations and the small spatial scale at which the occurrence of TBPs may vary.

The most frequently detected pathogen was, expectedly, *B. burgdorferi* s.l., which was detected annually from both nymphs and adults. Indeed, a recent countrywide citizen science survey revealed that ~14% of nymph and 17% of adult *I. ricinus* and *I. persulcatus* in Finland carry *B. burgdorferi* s.l. and that the occurrence of the pathogens largely coincides with the distribution of these tick species^5^. However, the prevalence rates observed for *B. burgdorferi* s.l. in the current study were higher than both the averages reported in the Finnish citizen science survey and those observed more widely for *I. ricinus* in Scandinavia (12.9 ± 2.2% for nymphs and 21 ± 4% for adults)^32^, suggesting that conditions on the island are particularly suitable for the circulation of at least some *B. burgdorferi* s.l. genospecies.

Apart from *B. burgdorferi* s.l., particularly high prevalence rates were not observed for other TBPs on the island. In fact, low prevalence rates were observed for *Rickettsia* and *Ca*. N. mikurensis compared to other European studies (*Rickettsia*:^33–35^; *C*. N. mikurensis^36–39^). Whereas *Rickettsia* spp. were detected from the island annually, *Ca*. N. mikurensis was not detected until 2015, 3 years into the study. As such, these results suggest that the pathogen may have emerged in the island’s tick population within the past few years. While low pathogen prevalence rates may generally reflect poor habitat quality regarding the circulation and upkeep of the pathogen, in this case, the observation might be explained by the potentially recent arrival of the pathogen on the island. Consequently, this highlights a problem that may arise when interpreting results from pathogen screenings without sufficient longitudinal data. Namely, whereas environmental factors may indeed explain low prevalence of a pathogen, recent establishment in the study area may also be the cause. For instance, had we conducted a single year pathogen screening in Seili in 2015, measured various environmental factors on the island, and related them to the observed low *Ca*. N. mikurensis prevalence, we might have inadvertently assigned environmental factors that are in reality linked to high habitat quality for the pathogen as indicators of poor habitat quality, as we would have no notion of the potentially recent arrival of the pathogen on the island. Such errors may be particularly misleading if the data are later used for projection in wide-scale predictive mapping of pathogen distribution^28^. Consequently, longitudinal studies of TBP occurrence at specific localities are required to diminish the chances of incorrect association of environmental attributes and TBPs.

TBEV, the subject of major health care interest, was likewise not detected until 4 years into the study, in 2016–17, suggesting recent emergence in the island’s tick population. Interestingly, most of the positive samples (3/3 in 2016, 2/4 in 2017) were found from an ecologically distinct area on the island, from transects within or in very close proximity to a grove of common hazel (*Corylus avellana*). Whereas different climatic factors may dictate where current and future TBEV foci are situated on a large spatial grain (^40–42^; however, also see refs ^43,44^), climatic differences were negligible between the study sites of the island, situated at maximum 1.3 km from each other. As such, to explain this phenomenon, we must consider differences between local microhabitats. The grove of common hazel is characterized by largely missing ground floor vegetation (ground floor comprised mostly of leaf litter), high canopy cover 2–5 m above the ground (potentially offering increased protection from avian predators for rodents), and hazelnuts (food for rodents). As such, it may offer a particularly good environment for certain rodent populations. Furthermore, as the questing of all tick life stages in the grove is forced to take place on top of leaf litter due to the lack of ground floor vegetation, high amounts of larvae and nymphs may encounter the same rodent hosts. Consequently, as the amplification and upkeep of TBEV in nature require co-feeding of larvae and nymphs on such hosts^45^, the occurrence of TBEV in this specific area of the island may result from the unique attributes of the grove. Future ecological and serological surveys of the rodent and insectivore populations in the grove are planned to assess this hypothesis.

Overall, TBEV is commonly known to circulate in specific, patchily distributed *I. ricinus* populations in Finland, with prevalence rates between 0.5% and 2%^5,15,46,47^. Southwestern Finland (including the Åland Islands) is a particularly well-known TBEV risk area, which still constitutes a major region for human TBE cases in Finland, despite active vaccination campaigns in the area (26.3–75% of annually diagnosed TBE cases in Finland between 1995–2017; Finnish Infectious Disease Register). Overall, the numbers of annual TBE cases in Finland have been increasing in the 21st century, suggesting increased TBEV occurrence in Finnish tick populations. Therefore, spread of the pathogen to Seili is not unexpected, and the virus may even have originated from nearby islands or the mainland. As the emergence of novel TBEV foci amidst changing climates is becoming more common (at least in some regions)^5,15,47–49^, more precise knowledge regarding the environmental conditions facilitating the spread and subsequent establishment of the virus in novel localities may prove crucial in predicting the emergence of future foci^42,44,50^.

The protozoan pathogen *B. venatorum* (previously *Babesia* sp. EU1) was detected annually, effectively forming the first report of the pathogen from Finnish ticks. Only one other member of the genus, *B. microti*, has previously been reported from Finnish *I. persulcatus*^51^. The prevalence rates of *Babesia* reported from European *I. ricinus* are typically relatively low, but rates higher than 10% have also been reported, even for nymphs^52–54^. Overall, the occurrence and diversity of *Babesia* in Finnish ticks are poorly known and should be further investigated, particularly as co-infections of *Babesia* and the most common tick-borne pathogens, *B. burgdorferi* s.l., appear to be able to cause more severe diseases in humans^18^. A rare fatal case of babesiosis was previously reported from a co-infected person in Finland^17^.

Indeed, co-infections with two or more TBPs may cause unpredictable diseases in humans. In some cases, as with *B. burgdorferi* s.l. and *Babesia*, patient cases with concurrent infections have been documented. However, for many potential pathogen combinations, few data exist. In the current study, co-infections were detected in 1.8% of nymphs and 3.8% of adult ticks. Interestingly, these data revealed that a particularly high proportion of *Ca*. N. mikurensis detections were from nymph samples co-infected with *B. burgdorferi* s.l. In fact, the rate of co-infection observed in the current study was more than double the value expected from random co-occurrence. Similar trends regarding the co-occurrence of these two pathogens have also been detected in other recent studies^55–57^, and the findings seem to be linked to wild rodents (especially the bank vole, *Myodes glareolus*) as common reservoir hosts for both pathogens^55,58^. As such, these results suggest at least partly overlapping enzootic cycles for *Ca*. N. mikurensis and some *B. burgdorferi* s.l. genospecies. Furthermore, as *B. burgdorferi* s.l. prevalence on the island is relatively high, these observations further support the notion that the observed low *Ca*. N. mikurensis prevalence on the island is likely connected to recent arrival rather than poor habitat quality. While *Ca*. N. mikurensis infections mostly manifest in patients with underlying diseases^39^, little is known about the clinical outcome of co-infections of these two pathogens in humans. However, as data suggest that they have a particular affinity for co-infection in at least *I. ricinus*, further efforts should aim to increase surveillance of both ticks and human patients.

Interestingly, despite the negligible climatic variability between study sites on Seili Island, persistent differences in prevalence estimates among biotopes were found. The highest prevalence of *B. burgdorferi* s.l. in nymphs was detected in deciduous forests and meadows, whereas pastures exhibited the lowest prevalence. In contrast, pastures displayed the highest prevalence of *A. phagocytophilum* in nymphs, while forested habitats (deciduous/coniferous forests and alder thickets) had much lower prevalence. Furthermore, in some cases we were able to analyze differences in prevalence between transects in similar biotopes. Unfortunately, due to the manner of sampling, which led to varied sample sizes across transects, this was only possible for a few biotope and pathogen combinations. Where such analysis could be carried out, it revealed differences in prevalence estimates, even within biotopes. A trend similar to that observed for biotopes was observed for transects in coniferous forests: one transect had lower *Borrelia* prevalence but higher *A. phagocytophilum* prevalence than the other two transects.

These results should not be interpreted as depicting any major trends in the occurrence of pathogens in different biotopes but rather as an indication that, even within the confines of a small island, biotopes, and individual transects in the same biotope class can differ from each other in regard to pathogen occurrence. As such, they emphasize the small scale at which local environmental factors may potentially affect the spatial distribution of pathogens. In a recent study, Boehnke et al.^59^ found that humidity can vary between open areas and forests as well as within different layers of a forest, directly affecting questing conditions for ticks. Indeed, the same is likely true for various other variables affecting tick questing and living conditions in different biotopes and structures [e.g., ground floor temperatures in shaded forests vs. open meadows (biotope type), different levels of canopy cover and wind shelter based on tree density (forest structure)]^60^. In this regard, the biotope classification applied in the current study, as well as similar classifications often used in tick-related studies, provide only rough estimations of in situ conditions for ticks and associated pathogens. Consequently, such classifications may fail to identify important differences influencing tick and pathogen occurrence across space. Further focus should be placed on more precise measurements of abiotic and biotic factors associated with the realized occurrence of ticks and tick-borne pathogens. By closely examining and identifying variables specifically affecting the occurrence of each individual pathogen, we may gain further insight into the factors driving their spread and circulation in tick populations. Consequently, such data could lead to more precise mapping of tick and pathogen occurrence—both known and emerging—which is required to prevent human infections.

## Materials and methods

Field surveys were conducted annually from May to September 2012–2017 on Seili Island in southwestern Finland (coordinates 60°14′4′′N, 21°57′7′′E), known as an *I. ricinus* and LB hotspot^6,13,31^. The details on tick collection and identification, DNA/RNA extraction, and qPCR-based pathogen screening (bacterial pathogens *B. burgdorferi* s. l., *B. miyamotoi*, *A. phagocytophilum*, *Rickettsia* spp., *C*. N. mikurensis, *Bartonella* spp., protozoan parasites *Babesia* spp., and TBEV) are described in the Technical Appendix.

**Fig. 3:**
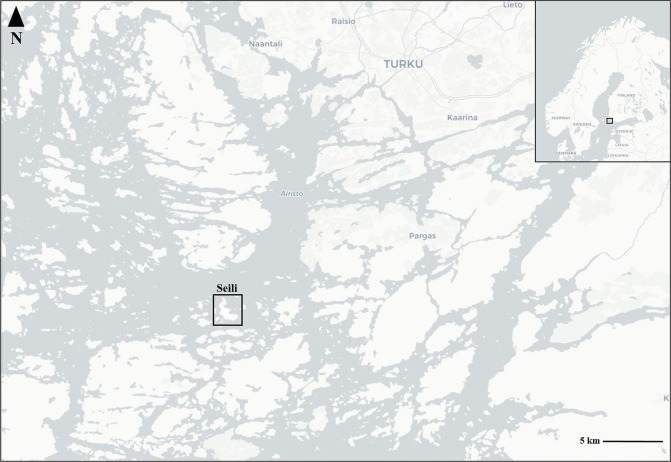
Study location. The location of the study area, Seili Island, in the Archipelago Sea, SW Finland

**Fig. 4:**
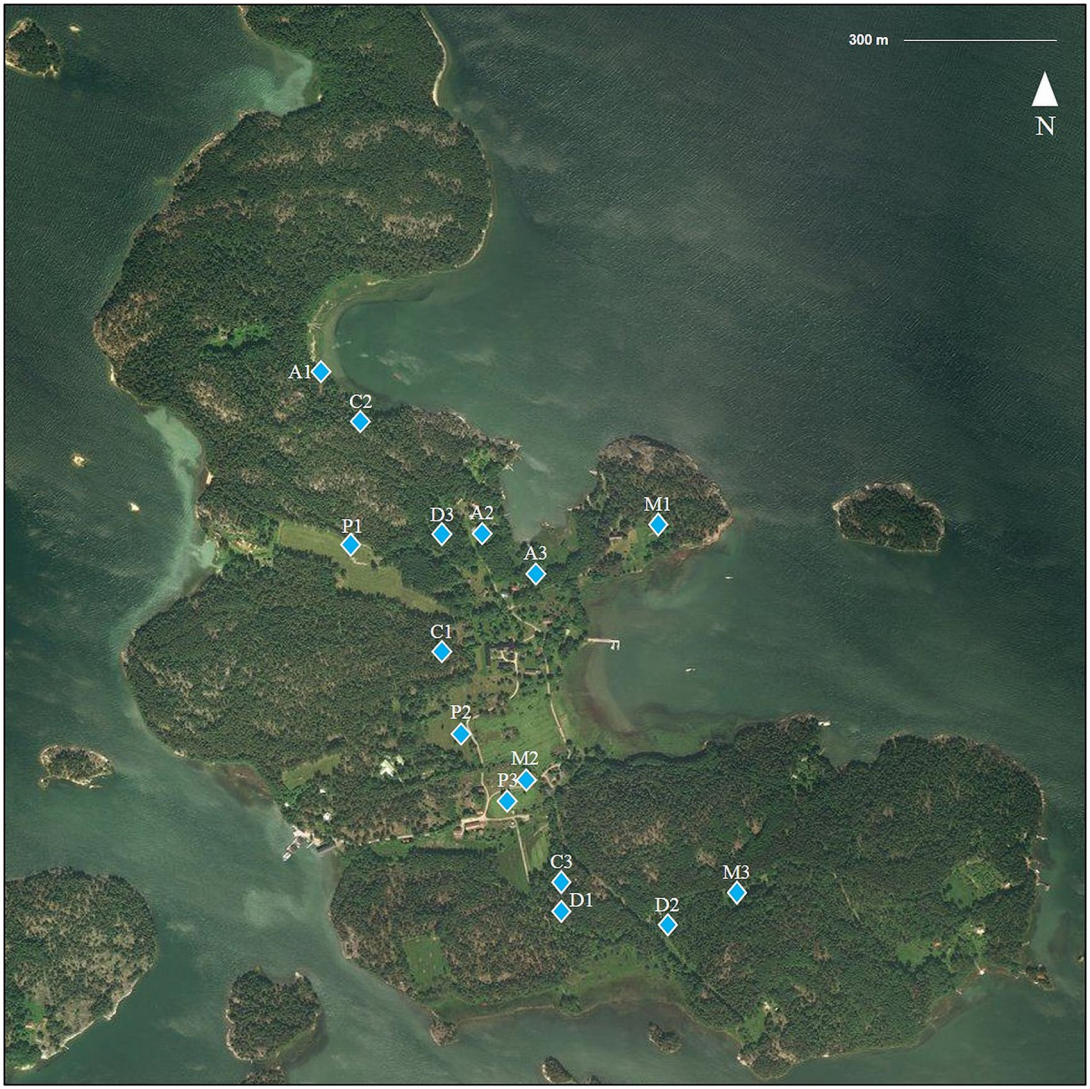
Study transects on Seili Island. Three transects were assigned to each biotope type, denoted with letter and number combinations. C coniferous forest, D deciduous forest, A alder thicket, M meadow, P pasture. The Archipelago Research Institute is located in the middle of the island

Several suitable host species for *I. ricinus* are known to inhabit the island: roe deer (*Capreolus capreolus*; approximately 20 individuals), white-tailed deer (*Odocoileus virginianus*; ~15 individuals), raccoon dogs (*Nyctereutes procyonoides*; several dozen), European hares (*Lepus europaeus*; no populations size estimates), and several species of ground-feeding birds (*Turdus merula*, *T. philomelos*, *T. pilaris*, *Erithacus rubecula*; several breeding pairs). Several species of rodents and insectivores also inhabit the island, but estimates of their numbers or diversity are not available. Seili lies relatively close to nearby islands (Figs. 3, 4), facilitating travel to and from the island by swimming for large mammals (roe deer, white-tailed deer, elks, raccoon dogs) during the summer and even for smaller animals during the winter, when the island is enclosed in ice. Naturally, for both migrating and local birds, the island is easily accessible, although low numbers of migrating birds are reported from the island (apart from those that nest there). In addition to these wild animals, cattle and sheep are brought to the island each spring, and they graze rotationally on pastures all around the island over the summer. Cattle have been brought to the island since 2008 and sheep since 2016.

### Statistical analyses

The probability of nymphs being positive for the analyzed pathogen in different biotopes (coniferous forest, deciduous forest, alder thicket, meadow, and pasture) was modeled by a generalized linear mixed model (GLMM), with binary error distribution and logit link function. Temporal arrangements of tick sampling were controlled for as random effects (year and month nested within year). Interactions between fixed (i.e., biotope) and random effects were not analyzed, as these models did not converge. Estimates for random effects (year) were predicted by the BLUPs (best linear unbiased prediction) obtained from the models.

For five biotope and pathogen combinations (*B. burgdorferi* s.l. and *A. phagocytophilum* prevalence among transects in coniferous forests, and *B. burgdorferi* s.l., *Rickettsia*, and *Ca*. N. mikurensis prevalence among transects in deciduous forest), it was also possible to analyze intra-biotope differences in prevalence. These GLMMs were otherwise similar to that described above, but the fixed explanatory factor was transect instead of biotope. Random effects were year and month nested within year. For *Ca*. N. mikurensis, only data from 2015–2017 were included in the analysis.

## Electronic supplementary material

Technical appendix
