# Supplementary material for: The importance of study duration and spatial scale in pathogen detection—evidence from a tick-infested island
Source: Emerg Microbes Infect. 2018 Nov 28;7:189. doi: 10.1038/s41426-018-0188-9 (PMC6258729; doi:10.1038/s41426-018-0188-9)
Supplement: Supplementary file 1 — Technical appendix [file 41426_2018_188_MOESM1_ESM.docx]

**The importance of study duration and spatial scale in pathogen detection – Evidence from a tick-infested island**

Technical appendix: Full laboratory protocols and statistical analyses

[Text A1: Additional methods 1](#_Toc508055916)

[Text A2: Additional information on real-time qPCR 2](#_Toc508055917)

[Text A3: Sequencing of positive samples 3](#_Toc508055918)

[Text A4: Additional information on statistical analyses 4](#_Toc508055919)

**Text A1: Additional methods**

Study locality

Field surveys were conducted annually from May to September in 2012-2017 on Seili Island. Seili is a relatively small rural island, located in the Archipelago Sea in southwestern Finland (surface area 1.6 km^2^; coordinates 60°14’4”N, 21°57’7”E). Rural landscapes on the island are partially managed by rotationally grazing cattle and sheep. Among locals it has long been known as an *I. ricinus* and LB hotspot. Indeed, it has been proven as such in studies conducted in the 21^st^ century [*1-3*].

Tick collection and sample preparation

Five different biotopes commonly found on the island were chosen for tick sampling: coniferous forest, deciduous forest, alder thicket, meadow, and pasture (biotopes described in more detail in [*2*]). Three 50 m study transects were placed in each biotope, for a total of 15 fixed transects. Transects within biotopes were chosen from different areas on the island, so that they were spatially separate. Ticks were sampled by dragging a 1 m^2^ cotton cloth along each transect 1-4 times per month from May to September. All ticks attached to the cloth were collected using tweezers. Tick species was determined either morphologically prior to extraction following the guidelines by Hillyard (1996), or using a duplex qPCR-assay designed to differentiate *I. ricinus* and *I. persulcatus*, as described before [*3*].

The biotopes were classified as follows: coniferous forests were forests dominated by *Picea abies* and *Pinus sylvestris*, with field layers clearly dominated by *Vaccinium myrtillus* and ground layers by mosses and needle litter. Deciduous forests were forests dominated by *Betula pendula* and *Betula pubescens* (*Corylus avellana* in transect D1) with field layers dominated by grasses and ground layers with patches of mosses, *V. myrtillus*, and leaf litter. Alder thickets were forested wetlands dominated by *Alnus glutinosa* with diverse field layers mostly dominated by *Filipendula ulmaria* and ground layers of mixed litter. Meadows were treeless areas with field layers of true grasses, mostly *Dactylis* sp., *Festuca* sp., and *Deschampsia* sp. Pastures were fenced grazing areas with flora similar to meadows. Each of the chosen pasture areas was grazed annually by cattle.

Total DNA and RNA were extracted from the frozen tick samples during 2013-2017 using NucleoSpin^®^ RNA and NucleoSpin^®^ 96 RNA –kits, and RNA/DNA buffer sets (Macherey-Nagel, Germany), following the kit protocols (RNA Kit: Rev. 16/May 2014; 96 RNA kit: Rev. 05/April 2014; RNA/DNA buffer set: Rev. 08/May 2014). RNA extracts were stored at -80 °C for later use. DNA extracts were stored at -20 °C.

**Text A2: Additional information on real-time quantitative PCR**

Real-time quantitative PCR (henceforth abbreviated qPCR) assays were carried out using SensiFAST™ Probe Lo-ROX Kit (for DNA) and SensiFAST™ Probe Lo-ROX One-Step Kit (for RNA) (Bioline, Germany). All DNA/RNA samples were analyzed in three replicate reactions carried out on 384-well plates. At least three blank water samples were used as negative controls in each assay. Samples were considered positive only when successful amplification was detected in all three replicate reactions.

DNA samples were screened for bacterial pathogens *Borrelia burgdorferi* sensu lato, *Borrelia miyamotoi*, *Anaplasma phagocytophilum*, *Rickettsia* spp., *Candidatus* Neoehrlichia mikurensis and *Bartonella* spp., and for protozoan parasites *Babesia* spp. Furthermore, RNA samples were screened for tick-borne encephalitis virus (TBEV). Analyses regarding *Borrelia* were carried out on individual DNA samples. For the screening of TBEV, *Rickettsia*, *A. phagocytophilum*, *Babesia*, *C.* N. mikurensis, and *Bartonella*, DNA/RNA samples were pooled (10 samples per pool, 5 μl of each sample) due to low expected prevalence. Individual samples from positive pools were subsequently re-analyzed as needed.

Borrelia burgdorferi s.l. and *B. miyamotoi* were screened from samples as described previously (Table A1, A2) [*3*]. DNA samples of *B. burgdorferi* s.l. and *B. miyamotoi* confirmed by sequencing in a previous study were used as positive controls [*2*].

For *A. phagocytophilum*, *Babesia* spp., and *C.* N. mikurensis screening, we used a multiplex qPCR assay, followed by individual assays (Table A1, A2). DNA samples of *A. phagocytophilum* and *Babesia microti* extracted from the blood of infected voles were used as positive controls for *A. phagocytophilum* and *Babesia* (provided by Eva R. Kallio, University of Oulu). For *C.* N. mikurensis, we used samples previously found positive.

*Rickettsia* and *Bartonella* were screened from pooled DNA samples using a duplex qPCR assay as described previously (Table A1, A2) [*3*]. Patient strains of *Bartonella grahamii* and *B. quintana* (provided by Arto Pulliainen, University of Turku) were used as positive controls for *Bartonella*. For *Rickettsia,* we used a commercially available control (ref. MBC042; Vircell, Granada, Spain).

Finally, TBEV was screened from pooled RNA samples using primers developed by Schwaiger & Cassinotti [*11*] (Table A1, A2). Multiple TBEV RNA strains (provided by Anu Jääskeläinen, University of Helsinki) were used as positive controls.

qPCR assays

The thermal cycling profile used for analyses of DNA samples was 95°C for 5 minutes, then 50 cycles of 95°C for 10 sec and 60°C for 30 sec (annealing/extension temperature was 58°C in assays involving *Rickettsia*). For RNA samples, thermal cycling profile was 48°C for 10 minutes (reverse transcription) and 95°C for 2 minutes, then 50 cycles of 95°C for 5 sec and 60°C for 30 sec. Thermal cycling was carried out at Finnish Microarray and Sequencing Centre (FMSC, Turku, Finland) using QuantStudio 12K Flex Real-Time PCR System (Life Technologies Inc. [LTI], Carlsbad, CA). All qPCR results were analyzed using QuantStudio™ 12K Flex Software v.1.2.2 (LT1). See Table A2 for qPCR mastermix configurations.

A portion of the samples (from years 2012-2014) had previously been screened for *Borrelia*, *A. phagocytophilum*, *Rickettsia*, *Bartonella*, *C.* N. mikurensis, and TBEV [*2, 3, 14*], but this is the first analysis of the whole set of pathogens frequently studied as etiological tick-borne agents of human diseases in Europe. Furthermore, no analyses had previously been done for 2015-2017 samples.

**Text A3: Sequencing of the positive samples**

Samples found positive for *Rickettsia* and *Babesia* were subsequently amplified by conventional PCR and sequenced, in order to determine species. Samples positive for *Rickettsia* were amplified using conventional PCR primers (CS877f and CS1258r) targeting Rickettsia *gltA* gene (Table A1). For *Babesia*, primers Bab-F and Bab-R targeting 18s rRNA of *Babesia* were used (Table A1).

PCR assays and sequencing

*Rickettsia* PCR was carried out in 12.5 μl reaction volume containing 3 μl of DNA extract, 2.75 μl ddH2O, 6.25 μl MyTaq RedMix polymerase mix (product number BIO-25048, Bioline, England), 500 nM forward primer, and 500 nM reverse primer. Thermal cycling was performed with the following program: 95°C for 3 min, then 35 cycles of 95°C for 20 sec, 61°C for 30 sec, and 72°C for 1 min.

*Babesia* PCR was carried out in 12 μl reaction volume containing 4 μl of DNA extract, 1.04 μl ddH2O, 6 μl MyTaq RedMix, and 400 nM forward and reverse primers. Thermal cycling profile was: 95°C for 3 min, then 50 cycles of 95°C for 30 sec, 58°C for 20 sec, and 72°C for 1 min.

Water samples were used as blank controls in each PCR batch. Successful PCR products were purified by mixing 1 μl EXO I enzyme, 1 μl rSAP enzyme, 3 μl of ddH2O, and 5 μl of PCR product, after which the samples were first incubated 5 min at 37°C and then heated 10 min at 80°C. Purified samples were sent to Macrogen Inc. Europe (The Netherlands) for sequencing. The sequences were trimmed using Geneious version 6 [*15*] and run through BLAST ([www.ncbi.nlm.nih.gov/BLAST/](http://www.ncbi.nlm.nih.gov/BLAST/)). The trimmed sequences were then further compared to reference sequences of corresponding species, downloaded from GenBank (www.ncbi.nlm.nih.gov/genbank/), to ascertain species using the software Geneious Pro R6 [*16*].

**Text A4: Additional information on statistical analyses**

Statistical analyses on prevalence differences of *B. burgdorferi* s.l., *A. phagocytophilum*, and *Rickettsia* spp. among five investigated biotopes were performed on individual nymph samples (n=1932) only. Further analyses were hindered by low sample sizes of the adult and larval ticks and by low numbers of positives for other pathogens (*Babesia*, *C.* N. mikurensis, *B. miyamotoi*, and TBEV) that were also unevenly distributed across years and biotopes. Furthermore, nymph samples from 2012 were excluded from the analyses because most of them were pooled, providing minimum infection rate (MIR) estimates instead of prevalence.

All the generalized linear mixed models (GLMM) were run with the GLIMMIX procedure of SAS v. 9.4. using restricted maximum likelihood estimation [*17*]. The method by Kenward and Roger (2009) was chosen to adjust standard errors and denominator degrees-of-freedom for tests of the fixed factors [*18*]. Estimates for random effects (year) were predicted by their BLUPs (best linear unbiased prediction) obtained from the models (Figure 1).

References

1. Mäkinen J, Vuorinen I, Oksi J, Peltomaa M, He Q, Marjamäki M, et al. Prevalence of granulocytic *Ehrlichia* and *Borrelia burgdorferi* sensu lato in *Ixodes ricinus* ticks collected from Southwestern Finland and from Vormsi Island in Estonia. Acta Pathologica, Microbiologica et Immunologica Scandinavica. 2003;111:355-62.

2. Sormunen JJ, Klemola T, Vesterinen EJ, Vuorinen I, Hytönen J, Hänninen J, et al. Assessing the abundance, seasonal questing activity, and Borrelia and tick-borne encephalitis virus (TBEV) prevalence of Ixodes ricinus ticks in a Lyme borreliosis endemic area in Southwest Finland. Ticks Tick Borne Dis. 2016;7(1):208-15.

3. Sormunen JJ, Penttinen R, Klemola T, Hänninen J, Vuorinen I, Laaksonen M, et al. Tick-borne bacterial pathogens in southwestern Finland. Parasit Vector. 2016;9(1):1-10.

4. Hillyard PD. Ticks of North-West Europe. The Dorset Press, Dorchester. 1996.

5. Courtney JW, Kostelnik LM, Zeidner NS, Massung RF. Multiplex Real-Time PCR for Detection of Anaplasma phagocytophilum and Borrelia burgdorferi. J Clin Microbiol. 2004;42(7):3164-8.

6. Vayssier-Taussat M, Moutailler S, Michelet L, Devillers E, Bonnet S, Cheval J, et al. Next Generation Sequencing Uncovers Unexpected Bacterial Pathogens in Ticks in Western Europe. PLoS ONE. 2013; e81439.

7. Diaz MH, Bai Y, Malania L, Winchell JM, Kosoy MY. Development of a novel genus-specific real-time PCR assay for detection and differentiation of Bartonella species and genotypes. J Clin Microbiol. 2012;50.

8. Labruna MB, Whitworth T, Horta MC, Bouyer DH, McBride JW, Pinter A. Rickettsia species infecting Amblyomma cooperi ticks from an area in the State of São Paulo, Brazil, where Brazilian spotted fever is endemic. J Clin Microbiol. 2004;42.

9. Jahfari S, Fonville M, Hengeveld P, Reusken C, Scholte E-J, Takken W, et al. Prevalence of Neoehrlichia mikurensis in ticks and rodents from North-west Europe. Parasit Vector. 2012;5(1):74.

10. Radzijevskaja J, Paulauskas A, Rosef O. Prevalence of Anaplasma phagocytophilum and Babesia divergens in Ixodes ricinus ticks from Lithuania and Norway. Int J Med Microbiol. 2008;298:218-21.

11. Schwaiger M, Cassinotti P. Development of a quantitative real-time RT-PCR assay with internal control for the laboratory detection of tick borne encephalitis virus (TBEV) RNA. J Clin Virol. 2003;27(2):136-45.

12. Mediannikov OY, Sidelnikov Y, Ivanov L, Mokretsova E, Fournier PE, Tarasevich I. Acute tick-borne rickettsiosis caused by Rickettsia heilongjiangensis in Russian Far East. Emerg Infect Dis. 2004;10.

13. Georges K, Loria GR, Riili S, Greco A, Caracappa S, Jongejan F, et al. Detection of haemoparasites in cattle by reverse line blot hybridisation with a note on the distribution of ticks in Sicily. Vet Parasit. 2001;99(4):273-86.

14. Sormunen JJ, Penttinen R, Klemola T, Vesterinen EJ, Hänninen J. *Anaplasma phagocytophilum* in questing *Ixodes ricinus* ticks in southwestern Finland. Exp Appl Acarol. 2016;70(4):491-500.

15. Kearse M, Moir R, Wilson A, Stones-Havas S, Cheung M, Sturrock S, et al. Geneious Basic: An integrated and extendable desktop software platform for the organization and analysis of sequence data. Bioinformatics. 2012;28(12):1647-9.

16. Drummond AJ, Ashton B, Buxton S, Cheung M, Cooper A, Duran C, et al. Geneious vR6.: http://www.geneious.com/. 2011.

17. Stroup W. Generalized linear mixed models: modern concepts, methods and applications.: CRC Press, Boca Raton. 2013.

18. Kenward MG, Roger JH. An improved approximation to the precision of fixed effects from restricted maximum likelihood. Comput Stat Data An. 2009;53(7):2583-95.

Table A1. Primers and probes used in pathogen screening and species determination

| Primer/probe name | Primer/probe target | 5’ 🡪 3’ | Reference |
| --- | --- | --- | --- |
| qPCR: |  |  |  |
| Bb23Sf | *B. burgdorferi* 23S RNA | CGAGTCTTAAAAGGGCGATTTAGT | Courtney et al. 2004 |
| Bb23Sr | *B. burgdorferi* 23S RNA | GCTTCAGCCTGGCCATAAATAG |  |
| Bb23Sp | *B. burgdorferi* 23S RNA | [FAM]-AGATGTGGTAGACCCGAAGCCGAGTG-[BHQ1] |  |
|  |  |  |  |
| Bmi-F | *B. miyamotoi glpQ* | CACGACCCAGAAATTGACACA | Vayssier-Taussat et al. 2013 |
| Bmi-R | *B. miyamotoi glpQ* | GTGTGAAGTCAGTGGCGTAAT |  |
| Bmi-P | *B. miyamotoi glpQ* | [FAM]-TCGTCCGTTTTCTCTAGCTCGATTGGG-[BHQ1] |  |
|  |  |  |  |
| Bart-ssRA-F | *Bartonella* *ssRa* | GCTATGGTAATAAATGGACAATGAAATAA | Diaz et al. 2012 |
| Bart-ssRA-R | *Bartonella* *ssRa* | GCTTCTGTTGCCAGGTG |  |
| Bart-ssRA-P | *Bartonella ssRa* | [FAM]-ACCCCGCTTAAACCTGCGACG-[BHQ1] |  |
|  |  |  |  |
| Rspp-F | *Rickettsia gltA* | GAGAGAAAATTATATCCAAATGTTGAT | Labruna et al. 2004 |
| Rspp-R | *Rickettsia gltA* | AGGGTCTTCGTGCATTTCTT |  |
| Rspp-P | *Rickettsia gltA* | [CY5]-CATTGTGCCATCCAGCCTACGGT-[BHQ3] |  |
|  |  |  |  |
| CNeGroEL-F | *Ca.* N. mikurensis *groEL* | CCTTGAAAATATAGCAAGATCAGGTAG | Jahfari et al. 2012 |
| CNeGroEL-R | *Ca.* N. mikurensis *groEL* | CCACCACGTAACTTATTTAGCACTAAAG |  |
| CNeGroEL-P | *Ca.* N. mikurensis *groEL* | [FAM]-CCTCTACTAATTATTGCWGAAGATGTAGAAGGTGAAGC-[BHQ1] |  |
|  |  |  |  |
| ApMSP2F | *A.phagocytophilum Msp2* | ATGGAAGGTAGTGTTGGTTATGGTATT | Courtney et al. 2004 |
| ApMSP2R | *A.phagocytophilum Msp2* | TTGGTCTTGAAGCGCTCGTA |  |
| ApMSP2P | *A.phagocytophilum Msp2* | [CY5]-TGGTGCCAGGGTTGAGCTTGAGATTG-[BBQ650] |  |
|  |  |  |  |
| Bab18S-F | *Babesia* 18S rRNA | CAGCTTGACGGTAGGGTATTGG | Radzijevskaja et al. 2008 |
| Bab18S-R | *Babesia* 18S rRNA | TCGAACCCTAATTCCCCGTTA |  |
| Bab18S-P | *Babesia* 18S rRNA | [HEX]-CGAGGCAGCAACGG-[BHQ1] |  |
|  |  |  |  |
| TBE1-F | TBEV non-struct. prot. 5 | GGGCGGTTCTTGTTCTCC | Schwaiger & Cassinotti 2003 |
| TBE1-R | TBEV non-struct. prot. 5 | ACACATCACCTCCTTGTCAGACT |  |
| TBE1-P | TBEV non-struct. prot. 5 | [FAM]-TGAGCCACCATCACCCAGACACA-[BHQ1] |  |
| PCR: |  |  |  |
| CS877f | *Rickettsia gltA* | GGGGACCTGCTCACGGCGG | Mediannikov et al. 2004 |
| CS1258r | *Rickettsia gltA* | ATTGCAAAAAGTACAGTGAACA |  |
|  |  |  |  |
| Bab-F | *Babesia* 18S rRNA | GACACAGGGAGGTAGTGACAAG | Georges et al. 2001 |
| Bab-R | *Babesia* 18S rRNA | CTAAGAATTTCACCTCTGACAGT |  |

Table A2. Mastermix contents for qPCR screening of pathogens

| qPCR target | Single/  duplex/  multiplex | Sample(s) | Volume | Probe mix^*^ | ddH2O | Forward/reverse primer concentration^†^ | Probe concentration^†^ | DNA/RNA template |
| --- | --- | --- | --- | --- | --- | --- | --- | --- |
| *B. burgdorferi* s.l. | Single | Single | 5 µl | 2.5 µl | 1.25 µl | 200 nM | 100 nM | 1 µl |
| *B. miyamotoi* | Single | Single | 5 µl | 2.5 µl | 1.25 µl | 200 nM | 100 nM | 1 µl |
| *Rickettsia*  *Bartonella* spp. | Duplex  Duplex | Pooled | 8 µl | 4 µl | - | 300 nM  200 nM | 150 nM  100 nM | 3 µl |
| *Rickettsia* | Single | Single | 5 µl | 2.5 µl | 1.25 µl | 200 nM | 100 nM | 1 µl |
| *Bartonella spp.* | Single | Single | 5 µl | 2.5 µl | 1.25 µl | 200 nM | 100 nM | 1 µl |
| *A. phagocytophilum*  *Babesia* spp.  *C.* N. mikurensis | Multiplex  Multiplex  Multiplex | Pooled | 10 µl | 5 µl | - | 300 nM  300 nM  185 nM | 150 nM  150 nM  125 nM | 3 µl |
| *A. phagocytophilum* | Single | Single | 5 µl | 2.5 µl | 1.25 µl | 200 nM | 100 nM | 1 µl |
| *Babesia* spp. | Single | Single | 5 µl | 2.5 µl | 1.25 µl | 200 nM | 100 nM | 1 µl |
| *C.* N mikurensis | Single | Single | 5 µl | 2.5 µl | 1.25 µl | 200 nM | 100 nM | 1 µl |
| TBEV | Single | Pooled | 8 µl | 4 µl | 0.13 µl | 400 nM | 100 nM | 3 µl |
| TBEV | Single | Single | 5 µl | 2.5 µl | - | 400 nM | 100 nM | 1 µl |

^*^For DNA samples: SensiFAST™ Probe Lo-ROX Kit; For RNA samples: SensiFAST™ Probe Lo-Rox One-Step Kit.

^†^See Table A1 for primer/probe sequences.

Figure 1. Inter-annual estimates of nymph prevalence for *B. burgdorferi* s.l., *A. phagocytophilum*, and *Rickettsia*. Estimates of annual prevalence were predicted by their BLUPs (best linear unbiased prediction) obtained from the GLMMs.
